# PacBio single molecule long-read sequencing provides insight into the complexity and diversity of the *Pinctada fucata martensii* transcriptome

**DOI:** 10.1186/s12864-020-06894-3

**Published:** 2020-07-13

**Authors:** Hua Zhang, Hanzhi Xu, Huiru Liu, Xiaolan Pan, Meng Xu, Gege Zhang, Maoxian He

**Affiliations:** 1 CAS Key Laboratory of Tropical Marine Bio-resources and Ecology, Guangdong Provincial Key Laboratory of Applied Marine Biology, South China Sea Institute of Oceanology, Chinese Academy of Sciences, Guangzhou, 510301 China; 2 University of Chinese Academy of Sciences, Beijing, 100049 China

**Keywords:** *Pinctada fucata martensii*, PacBio sequencing, Alternative splicing, LncRNAs, Differentially expressed transcripts

## Abstract

**Background:**

The pearl oyster *Pinctada fucata martensii* is an economically valuable shellfish for seawater pearl production, and production of pearls depends on its growth. To date, the molecular mechanisms of the growth of this species remain poorly understood. The transcriptome sequencing has been considered to understanding of the complexity of mechanisms of the growth of *P. f. martensii.* The recently released genome sequences of *P. f. martensii*, as well as emerging Pacific Bioscience (PacBio) single-molecular sequencing technologies, provide an opportunity to thoroughly investigate these molecular mechanisms.

**Results:**

Herein, the full-length transcriptome was analysed by combining PacBio single-molecule long-read sequencing (PacBio sequencing) and Illumina sequencing. A total of 20.65 Gb of clean data were generated, including 574,561 circular consensus reads, among which 443,944 full-length non-chimeric (FLNC) sequences were identified. Through transcript clustering analysis of FLNC reads, 32,755 consensus isoforms were identified, including 32,095 high-quality consensus sequences. After removing redundant reads, 16,388 transcripts were obtained, and 641 fusion transcripts were derived by performing fusion transcript prediction of consensus sequences. Alternative splicing analysis of the 16,388 transcripts was performed after accounting for redundancy, and 9097 gene loci were detected, including 1607 new gene loci and 14,946 newly discovered transcripts. The original boundary of 11,235 genes on the chromosomes was corrected, 12,025 complete open reading frame sequences and 635 long non-coding RNAs (LncRNAs) were predicted, and functional annotation of 13,482 new transcripts was achieved. Two thousand three hundred eighteen alternative splicing events were detected. A total of 228 differentially expressed transcripts (DETs) were identified between the largest (L) and smallest (S) pearl oysters. Compared with the S, the L showed 99 and 129 significantly up-and down-regulated DETs, respectively. Six of these DETs were further confirmed by quantitative real-time RT-PCR (RT-qPCR) in independent experiment.

**Conclusions:**

Our results significantly improve existing gene models and genome annotations, optimise the genome structure, and in-depth understanding of the complexity and diversity of the differential growth patterns of *P. f. martensii*.

## Background

*Pincata fucata martensii* is one of the most common oysters used for the production of seawater pearls, food and drugs. It is also one of the most useful animals for studying biominerals, hence it is often used as a model system to investigate the molecular basis of biomineralisation [1, 2]. The growth, yield and quality of *P. f. martensii* is affected by various exogenous and endogenous factors, such as food availability [3], ocean acidification [4], temperature [5] and others. In recent years, increased mortality and slow growth have caused a distinct decline in pearl production due to a worsening aquaculture environment and aquatic diseases [6, 7]. However, limited information exists on the molecular mechanisms that regulate the growth and development of this species. In recent years, molecular approaches such as linkage maps [8], transcriptomics, and proteomics [9] have been applied to reveal growth traits and guide the molecular breeding of various bivalves. Thus, a comprehensive understanding of the mechanisms of growth and development is required to improve pearl production.

RNA sequencing (RNA-seq) has become a powerful technique for investigating gene expression profiles and revealing signal transduction pathways in a wide range of biological systems [10]. In the past few years, substantial effort has been invested in genetic and genomic research related to *P. f. martensii.* In particular, RNA-seq has yielded new information at both the transcriptome [11, 12] and genome [13, 14] level. RNA-seq has shaped our understanding of many aspects of biology, such as revealing the extent of mRNA splicing and the regulation of gene expression. Although the genome sequence of *P. f. martensii* has been completed recently [14], the gene structure still needs to be optimized and perfected. Due to the limitation of short sequencing reads, it is difficult to accurately predict full-length (FL) splice isoforms [15]. Additionally, the extent of alternative splicing (AS) and transcriptome diversity remains largely unknown. Recently, the Pacific Bioscience (PacBio) Single Molecule Real Time Sequencing (SMRT) technique can overcome the limitation of short read sequences, enabling the detection of novel or rare splice variants that are crucial for post-transcriptional regulatory mechanisms, and increasing transcriptome diversity and functional complexity [16–18]. The PacBio single-molecule approach eliminates the need for sequence assembly, facilitates the accurate elucidation of FL transcripts and primary-precursor-mature RNA structures, and provides a better understanding of RNA processing due to its ability to sequence reads up to 50 kb [17, 19]. However, PacBio sequencing also has its own limitations, such as high sequencing error rates and low throughput [20, 21]. Fortunately, PacBio sequencing and Illumina sequencing are highly complementary to each other [22]. To address these issues, we herein propose a hybrid sequencing strategy that can provide more accurate information and generate more data in terms of volume of *P. f. martensii* than either technique alone.

In shellfish, understanding the differences between individuals is very important for developing strategies in breeding. Screening for growth-related candidate genes has helped advance molecular genetics and breeding [23, 24]. Growth of oysters were regulated by a series of genes associated with protein synthesis, signal transduction and metabolism [9, 11]. Thus, identification of various differentially expressed genes involved in individual differences can provide insights into the growth mechanism, and develop suitable molecular markers for breeding [25]. Because growth mechanisms are complex and relate to many physiological processes, growth-related molecules derived from oysters have been studied using Illumina sequencing [11, 24, 26]. However, PacBio sequencing can provide further information on transcript diversity, including alternative splicing and alternative polyadenylation [15, 20]. Combined with PacBio sequencing and Illumina sequencing, more gene isoforms could be detected, revealing functional variety [18, 27].

In order to better explore the growth differences between largest and smallest pearl oyster groups, we performed PacBio sequencing and Illumina sequencing. The results may permit reannotation of the transcriptome, improve whole-genome annotation, optimise the genome structure, and provide a valuable genetic resource for further studies of pearl oysters growth.

## Results

### PacBio single molecule long-read sequencing data analysis

**Fig. 1:**
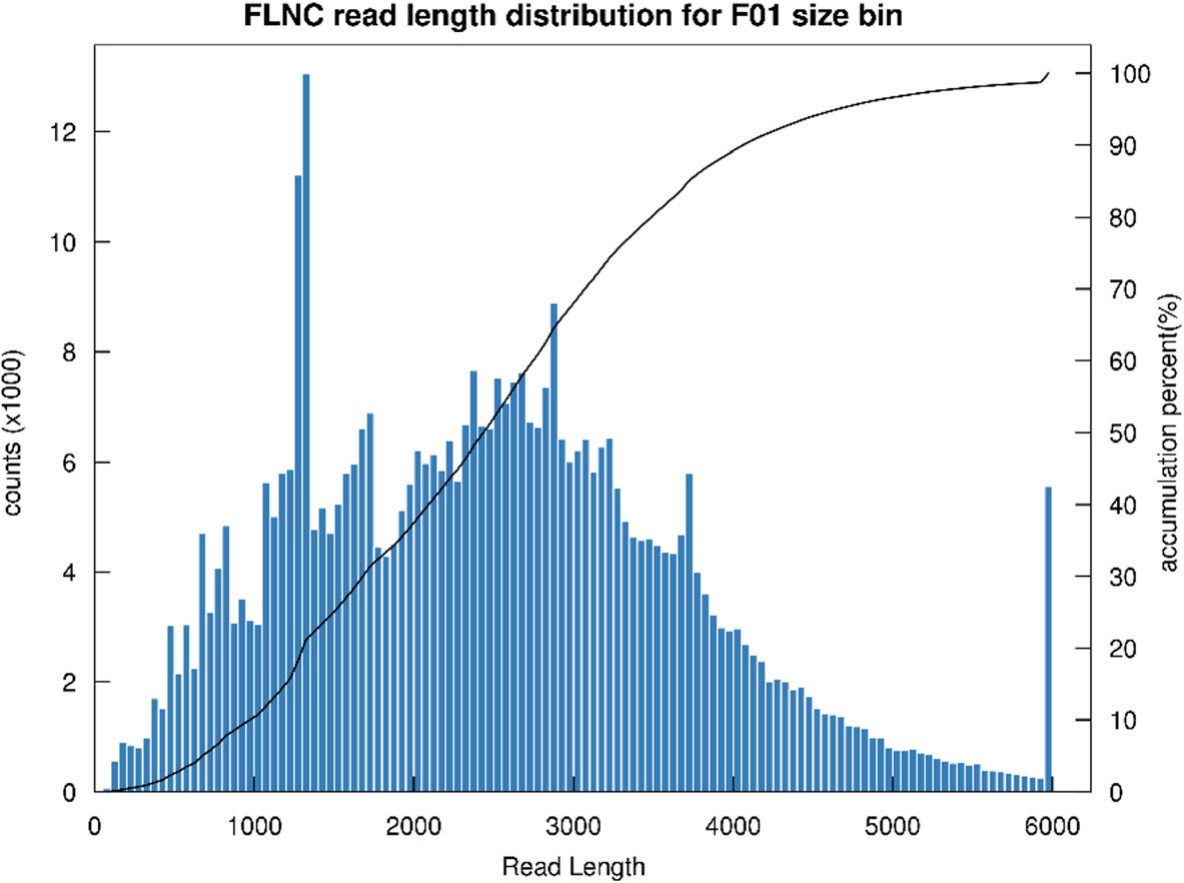
Density distribution of full length readsnon-chimeric (FLNC) read length obtained by SMART sequencing

Full-length cDNA sequences are important for correct annotation and identification of authentic transcripts from animal tissues. To generate a high quality transcriptome for *P. f. martensii*, we constructed 1–6 kb libraries and performed PacBio SMRT sequencing, which provides single-molecule, full-length transcript sequencing. A total of 2.65 Gb of clean reads were obtained. The Circular Consensus (CCS) library included 1,589,889,145 bp with a mean length of 2767 bp (Table 1). A total of 574,561 CCS reads were obtained after filtering with SMRTLink (4.0). In total, 54,400 high-quality isoforms were identified, with 443,944 full-length reads (77.27% of total CCS reads). In addition, 32,755 consensus isoforms were obtained, including 655 low-quality and 32,095 high-quality isoforms. The average consensus isoform read length was 2708 bp, and the density distribution of full length reads non-chimeric (FLNC) read length is shown in Fig. 1. Meanwhile, Illumina sequencing library was used to correct errors for further improve the accuracy of consensus reads. Using Illumina sequencing, 152 million paired-end reads were sequenced. We used Proovread [28] to correct the FLNC reads based on the Illumina sequencing. A total of 16,388 non-redundant transcripts were generated. BUSCO v3.0 (Benchmarking Universal Single Copy Orthologs) was utilized to determine completeness of our transcript dataset. The results showed that 41.3% (125 genes) were complete single-copy BUSCOs, 21.5% (65 genes) were complete duplicated BUSCOs, 6.6% (20 genes) were fragmented BUSCO archetypes, and 30.6% (93 genes) were missing BUSCOs entirely.

**Table 1:** The PacBio SMRT sequencing information of *P. f. martensii*

Category	Dataset
Read bases of Circular Consensus (CCS)	1,589,889,145
Number of ccs	574,561
Number of undesired primer reads	80,026
Number of undesired poly-A reads	363,918
Number of filtered short reads	398
Number of full-length non-chimeric reads	443,944
Full-length non-chimeric percentage (FLNC%)	77.27%
Number of consensus isoforms	32,755
Average consensus isoforms read length	2708
Number of polished high-quality isoforms	32,095
Number of polished low-quality isoforms	655
Gene loci	9097
New gene loci	1607

### Improving *P. f. martensii* genome annotation by PacBio sequencing

**Fig. 2:**
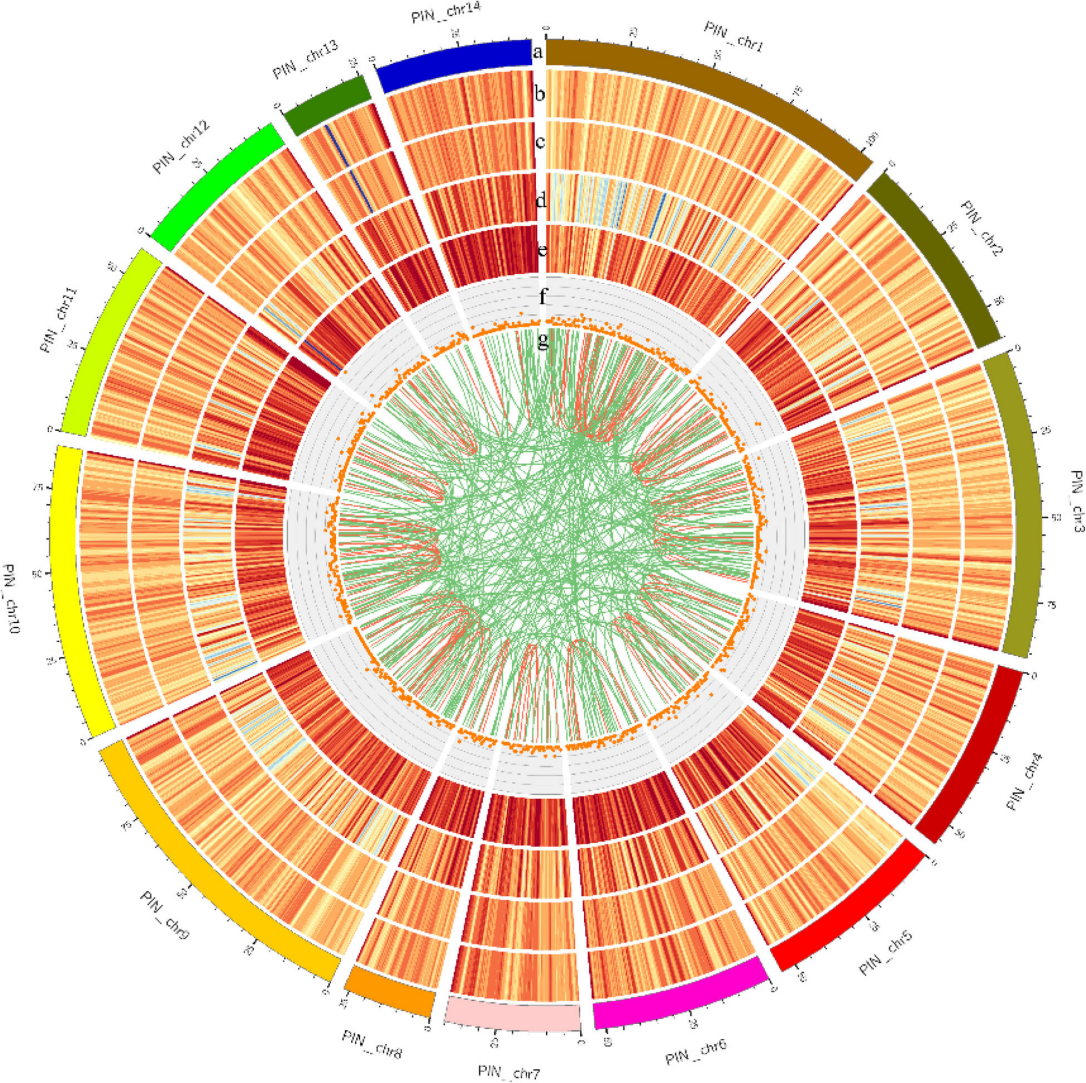
CIRCOS visualisation of the distribution of different data at the genome-wide level. **a**: *Pincata fucata martensii* chromosomes. **b**: Gene density of the reference genome. **c**: Density of genes predicted from the PacBio data. **d**: Transcript density in the genome. **f**: Long non-coding RNA (lncRNA) distribution in chromosomes. **g**: Fusion transcript distribution. Intra-chromosome data are coloured red inter-chromosome (green)

**Fig. 3:**
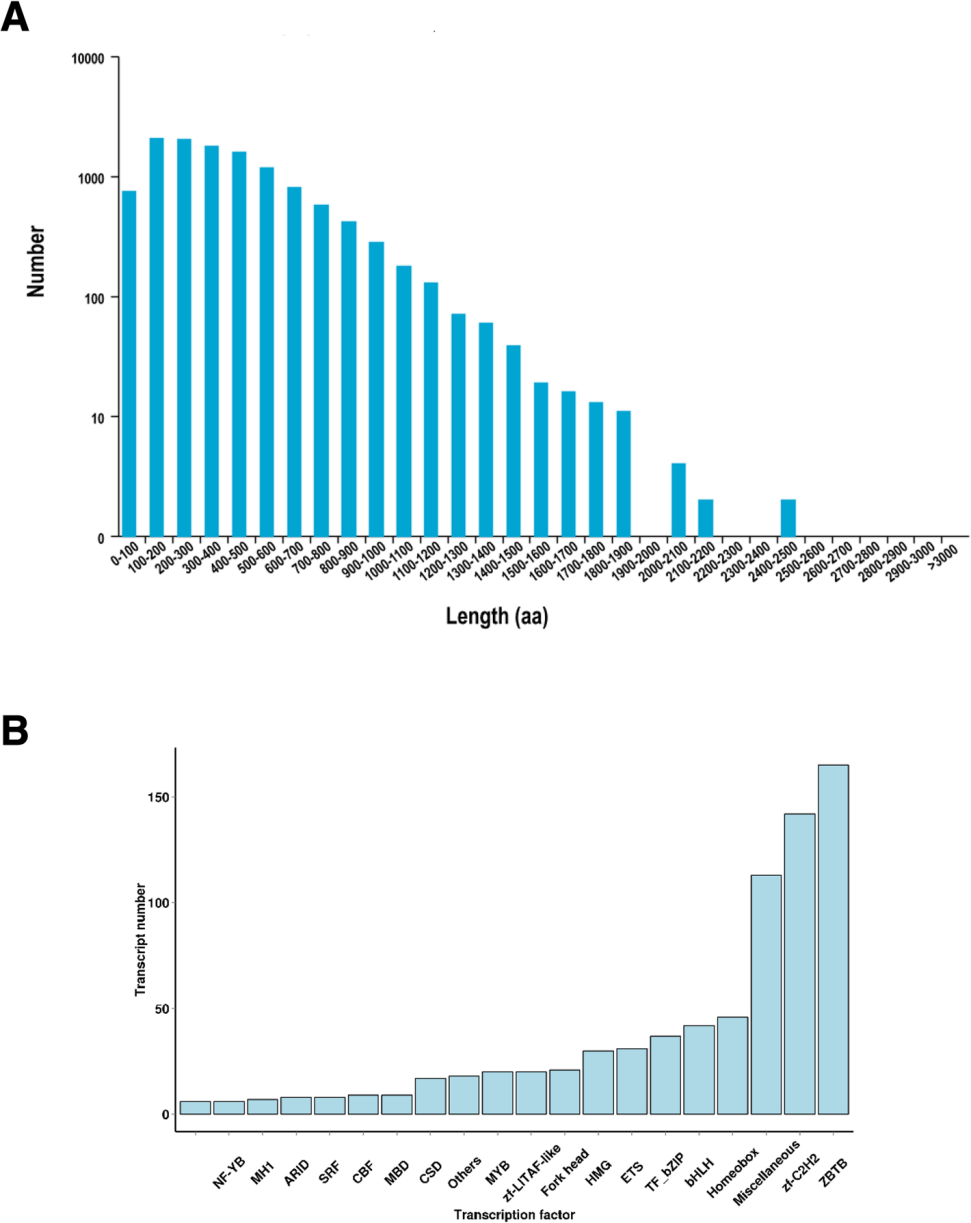
Length distribution of complete open reading frames (cds) (**a**) and type distribution of transcription factors (**b**)

Due to the limitations of the short read sequencing, annotation of the selected reference genome may not be sufficiently accurate, hence it is necessary to optimise the genetic structure of the original annotation. The PacBio technique has the advantage of sequencing length, and has been employed toward the optimisation of gene structure and the discovery of new transcript isoforms. The positions of 11,235 genes in the genome was optimised by the PacBio technique (Additional file 1: Table S1a, b), and 9097 gene loci were detected, of which 1607 were new gene loci. Gene fusion is caused by somatic chromosomal rearrangement, and fusion transcripts are related to the splicing machinery [29]. Herein, 641 fusion genes were identified in the PacBio library, and were validated using transcriptome datasets. The majority of these transcripts were mapped to the first and ninth chromosomes, but the location of 44 fusion genes was unknown (Additional file 2: Table S2a, b). The number of intra-chromosomal fusion transcripts was much lower than that of inter-chromosomal fusion genes in the circos map (Fig. 2). Coding region sequences and their corresponding amino acid sequences were analysed using TransDecoder software (v3.0.0) based on new transcripts obtained from AS. Comparison with the *P. f. martensii* genome identified 14,313 open reading frame (ORFs), of which 12,025 complete ORFs were generated by PacBio sequencing. Meanwhile, length distribution of the encoded protein sequence for each complete ORF region was mapped, and the results are shown in Fig. 3a. Transcription factors (TFs) are essential for regulation of gene expression. Based on the animalTFDB 2.0 database, 836 transcripts were predicted to be TFs. The main TFs identified in this work belong to the ZBTB, zf-C2H2, Miscellaneous, Homeobox and bHLH families (Fig. 3b).

### Putative molecular marker detection

Transcripts longer than 500 bp were screened to analyse SSR transcripts using the MIcroSAtellite identification tool (MISA). The total size of examined sequences was 44,854,919 bp, the total number of identified SSRs was 8061, and the number of SSR-containing sequences was 5303 from 16,127 FL transcripts. Perfect SSRs included 6366 mono-nucleotide SSRs, 936 di-nucleotide SSRs, 634 tri-nucleotide SSRs, 109 tetra-nucleotide SSRs, 15 penta-nucleotide SSRs and one hexa-nucleotide SSR. The number of SSRs gradually decreased with an increasing number of repeated SSR motifs. Mono-nucleotides showed the highest density. All SSRs are listed in Additional file 3: Table S3.

### Alternative polyadenylation (APA) and alternative splicing (AS) analysis

**Fig. 4:**
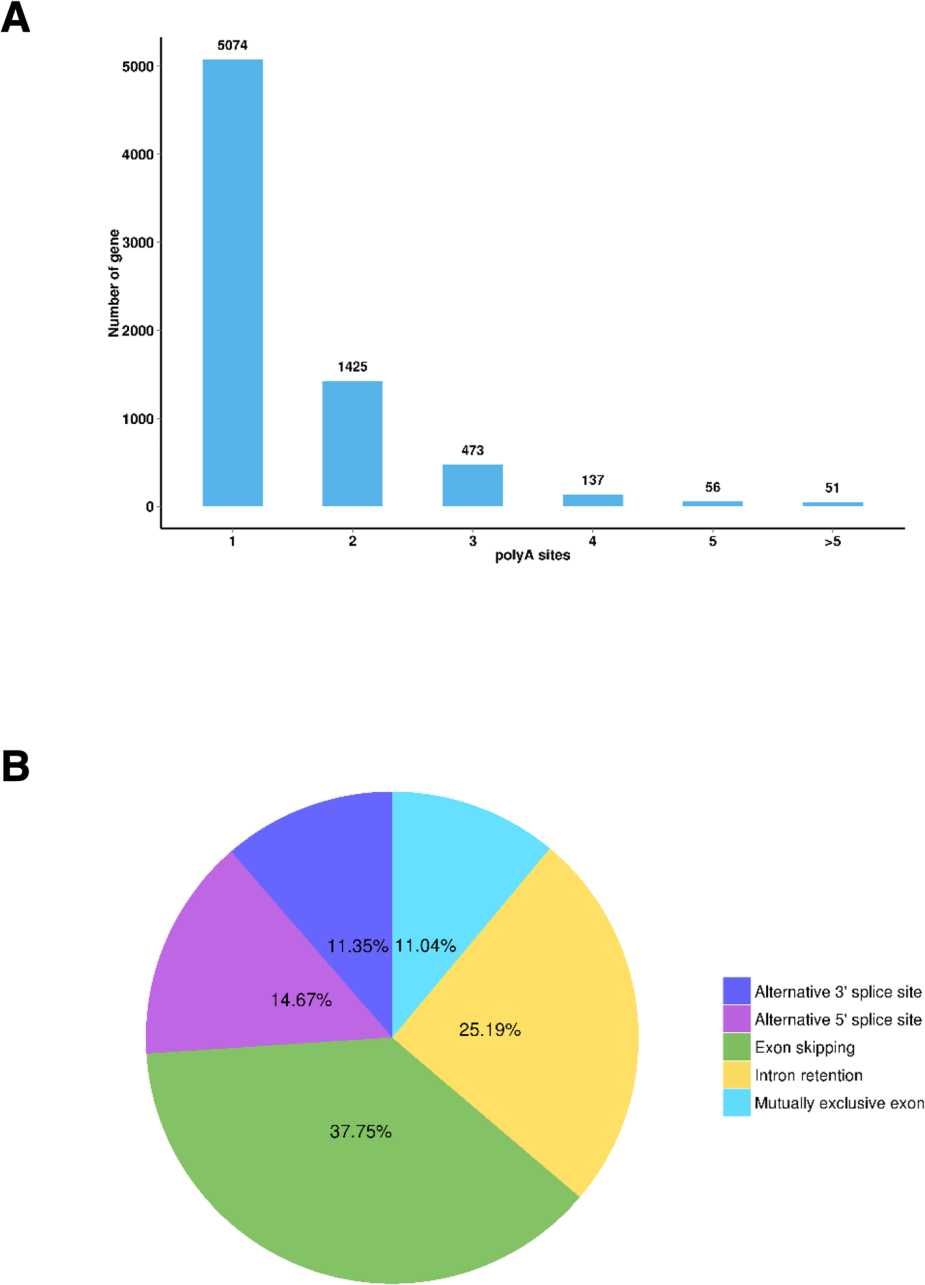
Characterisation of poly (A) sites and alternative splicing (AS) events. **a**: Distribution of the number of poly (A) sites per gene. **b**: Number of alternative splicing (AS) events

Polyadenylation is an important co-transcriptional modification in most eukaryotic transcripts. Alternative polyadenylation regulates gene expression and enhances the complexity of the transcriptome. A total of 7216 genes detected by the APIS pipeline have at least one poly (A) site, and 2142 genes have at least two or more poly (A) sites (Fig. 4a; Additional file 4: Table S4). Mature mRNAs are generated by a variety of splicing methods, and are translated into different proteins to increase biological complexity and diversity. The most important advantages of PacBio sequencing is its ability to identify AS events. A total of 2318 AS transcripts were predicted from the PacBio sequence data using AStalavista analysis, of which 177 AS transcripts were not annotated in the published version of the *P. f. martensii* genome (Additional file 5: Table S5a, b). Five kinds of AS events were identified (Fig. 4b); mutually exclusive exons (11.04%), intron retention (25.19%), exon skipping (37.75%), alternative 5′ splice sites (14.67%) and alternative 3′ splice sites (11.35%). Exon skipping and intron retention events were much more abundant than the other three types. The location of AS transcripts in the genome was described for all but 177 AS transcripts.

### Functional annotation of transcripts

**Fig. 5:**
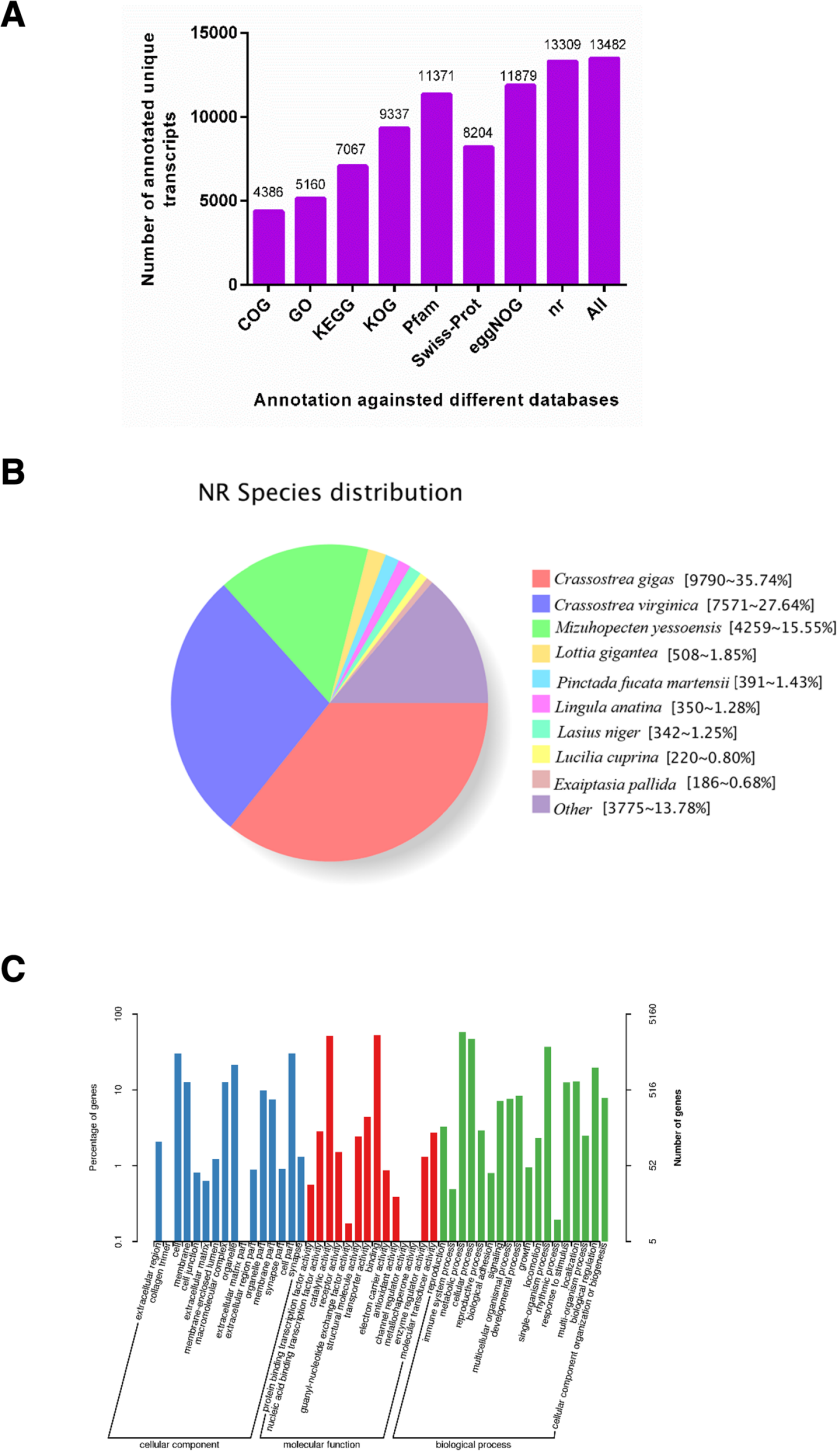
Functional annotation of non-redundant transcripts in public databases. **a**: Number of full-length transcripts annotated in COG, GO, KEGG Nt, Nr, SwissProt, KOG, and Pfam databases. **b**: GO classification of the putative functions of unique transcripts. **c**: Distribution of homologous species annotated in the NR database

The newly identified transcripts sequence were scanned against the NCBI non-redundant protein sequences (NR), Protein family (Pfam), Clusters of Orthologous Groups of proteins (KOG/COG/eggNOG), a manually annotated and reviewed protein sequence database (Swiss-Prot), Kyoto Encyclopedia of Genes and Genomes (KEGG) and Gene Ontology (GO) databases using BLAST 2.2.26 software to obtain annotation information for each transcript. The number of transcripts annotated in each database is shown in Fig. 5a. In total, 4386 transcripts were annotated in the COG database, 5160 were annotated in GO, 7067 were annotated in KEGG, 9337 were annotated in KOG, 11,371 were annotated in Pfam, 8204 were annotated in Swiss-Prot, 11,879 were annotated in eggNOG, and 13,309 were annotated in NR. Moreover, 13,482 transcripts were annotated in all databases. Meanwhile, new transcripts obtained from AS analysis were functionally annotated. Based on NR annotation, species homologous with *P. f. martensii* were predicted by sequence alignment. *Crassostrea gigas* and *Crassostrea virginica* were the closest matching genomes, followed by *Mizuhopecten yessoensis* (Fig. 5b). In GO annotation (Fig. 5c), transcripts were classified into three main GO categories; cellular component (CC), molecular function (MF) and biological process (BP). In the three main categories, metabolic process (BP) (4663), catalytic activity (MF) (4198) and cell part (CC) (2308) were the most enriched subcategories, respectively. Besides, the published version of *P.f.martensii* genome annotations contains 32,937 protein-coding gene models [14]. In the transcriptome database, 1028 gene are not annotated in the genome. To assess the presence of these unannotated genes, we conducted BLAST analyses, 516 were found in the blastx search against Swiss-Prot proteins, 986 in NR, 245 in COG database,309 in GO, 416 in KEGG, 578 in KOG,804 in eggNOG and 781 in Pfam (Additional file 6: Table S6).

### LncRNA prediction

**Fig. 6:**
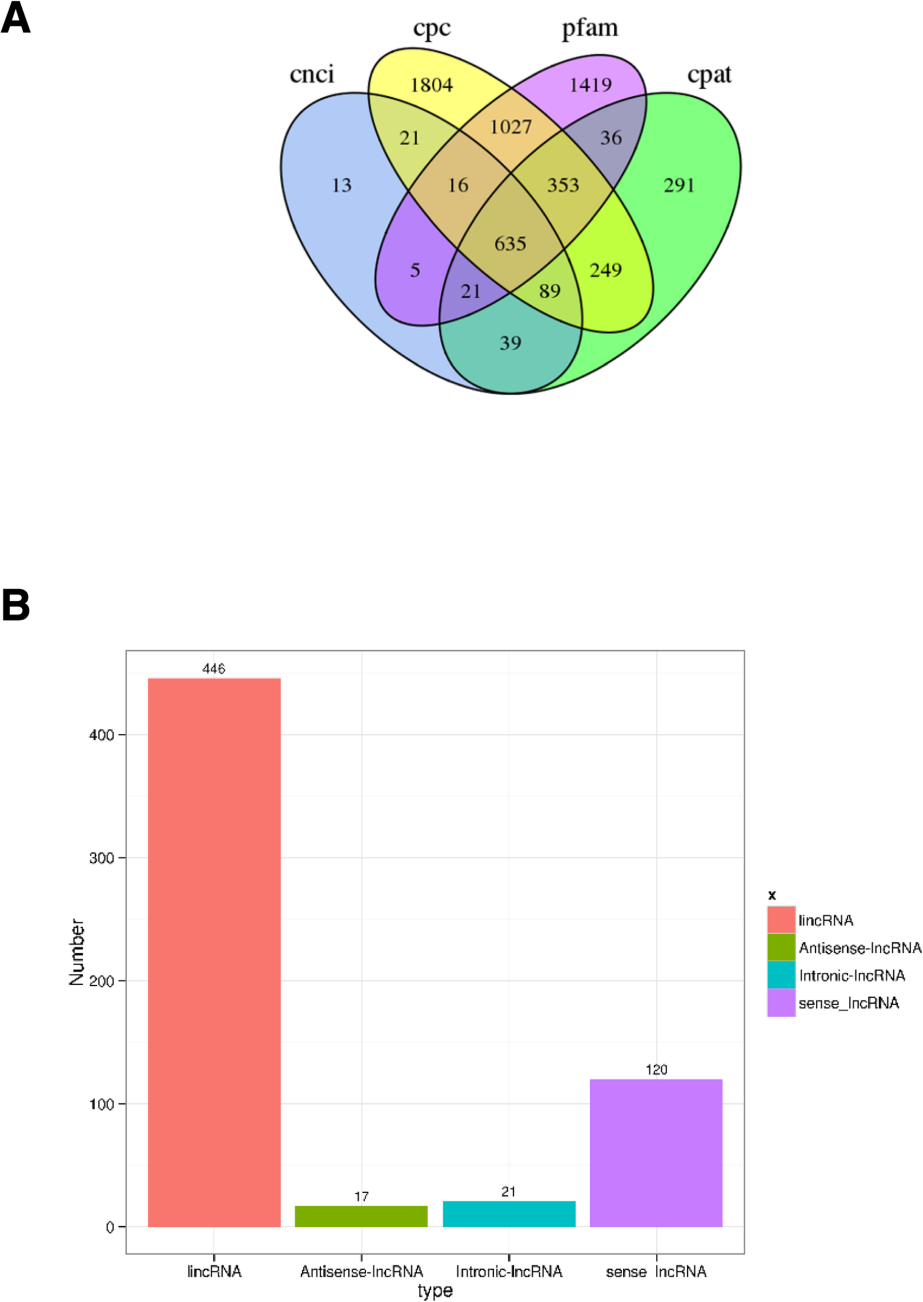
Prediction of lncRNAs in *Pincata fucata martensii*. **a**: Candidate lncRNAs identified using CNCI, CPC, Pfam and CPAT. Non-overlapping areas indicate the number of lncRNAs identified by the single tool; overlapping areas indicate the total number of lncRNAs identified by multiple tools. **b**: Types of lncRNAs

LncRNAs play an important role in regulating gene expression in most eukaryotes. Based on Coding Potential Calculator (CPC), Coding-Non-Coding Index (CNCI), Pfam protein structure domain and Coding Potential Assessment Tool (CPAT) analyses, the number of lncRNAs transcripts was 4194, 839, 3512, and 1713, respectively (Additional file 7: Table S7a, b), across all chromosomes. Additionally, 635 lncRNAs transcripts were identified in all analyses (Fig. 6a). Identification of lncRNAs was classified based on their position in the reference genome and annotation information. The 635 lncRNAs included 120 sense-lncRNAs, 21 intronic-lncRNAs, 17 antisense-lncRNAs and 446 lncRNAs (Fig. 6b). To investigate the functions of lncRNAs, we identified the potential targets of lncRNAs based on positional relationships between lncRNAs and mRNAs, and correlation analysis between lncRNAs and mRNA expression in samples (Additional file 8: Table S8). Mapping lncRNAs to chromosomes revealed that they have a distribution similar to that of mRNAs (Fig. 2).

### Differentially alternative splicing (AS) and differentially expressed transcripts (DETs) analysis

A single gene can generate functionally distinct mRNAs and diverse protein isoforms by recognition of exons and splice sites during splicing. We performed differentially variable splicing analysis between the L (L01, L02, L03 represent three subgroups from L groups) and S (S01, S02, S03 represent three subgroups from S groups) groups using RNA-seq. The expression correlation for S01 sample oysters was inconsistent with that of S02 and S03. Hence, data from the S01 sample were removed. Interestingly, the data showed that the number of the five basic types of AS models (except for A3SS in L groups) was much higher than for S groups; 144 significantly differential AS events in S groups were detected using junction counts alone, including 83 in SE, 44 in MEX, four in A5SS, three in A3SS and ten in RI. A total of 147 significantly differential AS events in L groups were identified using both junction counts and reads on targets, including 87 in SE, 42 in MEX, four in A5SS, five in A3SS and nine in RI. The number of AS events in L and S groups are shown in Additional file 9: Table S9.

**Fig. 7:**
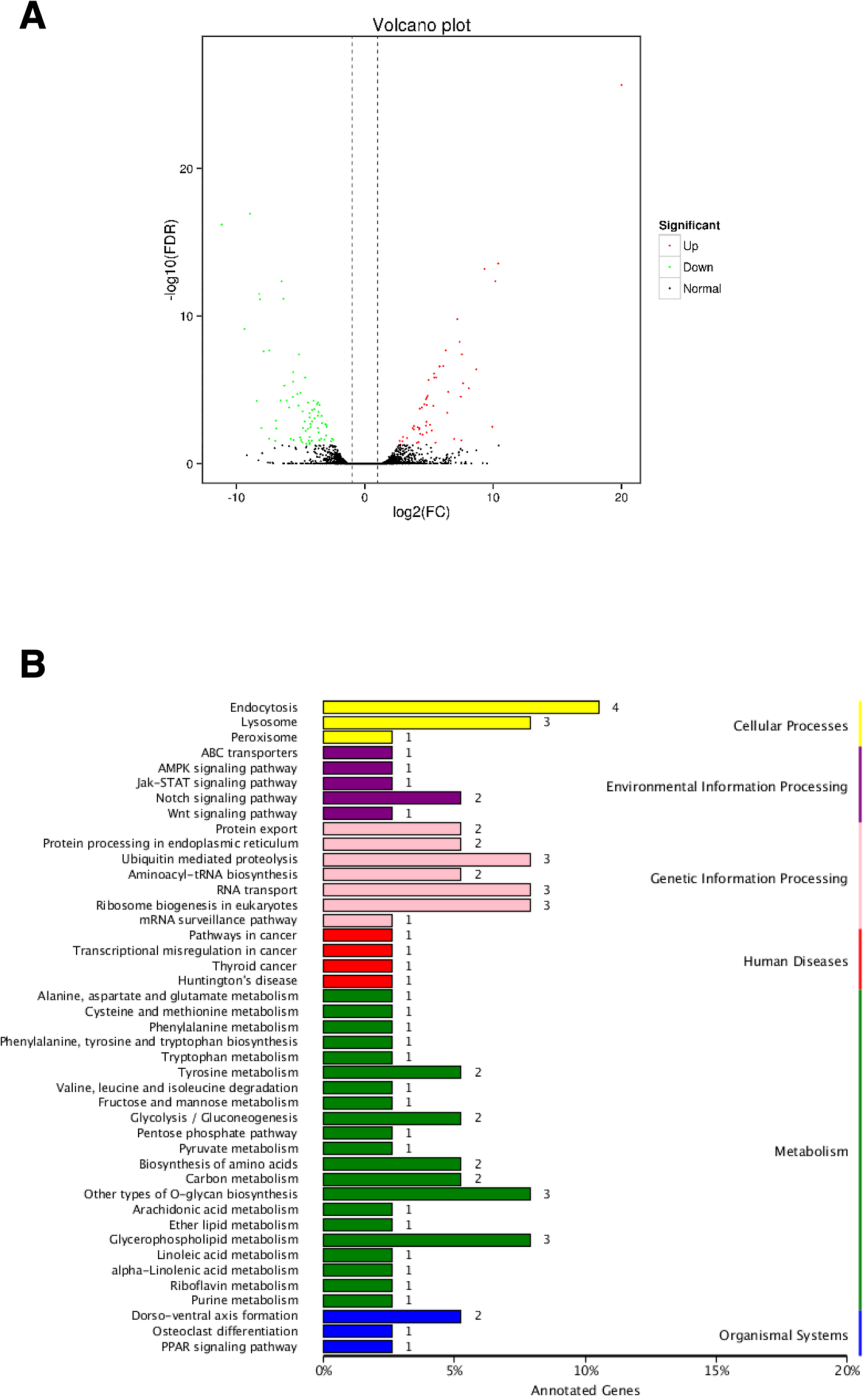
Analysis of DETs between S and L subgroups. **a**: volcano plot of transcripts. Each point in the volcano plot represents a transcript, the abscissa represents the logarithm of the difference in expression of a transcript in the two samples, and the ordinate represents the negative logarithm of the statistical significance of the change in the expression of the transcript. Green dots represent down-regulated DETs, red dots represent up-regulated DETs, and black dots represent non-DETs. **b**: KEGG classification map of DETs. The ordinate represents the KEGG metabolic pathway, and the abscissa represents the number of transcripts annotated to the pathway and the percentage of transcripts in the annotation

Transcript expression displays temporal and spatial specificity. Post-transcriptional processing of precursor mRNAs leads to transcript diversity, and hence diverse biological functions. We performed Illumina sequencing to search for transcripts shared between L and S groups. The FPKM method was used to estimate DETs. Our analysis yielded 228 DETs (|log2FC| ≥ 2, FDR < 0.01), among which 99 were up-regulated and 129 were down-regulated in the pairwise groups (Additional file 10: Table S10). Differences in expression levels of transcripts in the pairwise comparisons are shown in a volcano plot (Fig. 7a). Interestingly, KEGG pathway analysis showed that DETs were mainly assigned to metabolism, followed by genetic information processing, cellular processes, environmental information processing, human diseases, and organismal systems (Fig. 7b).

**Fig. 8:**
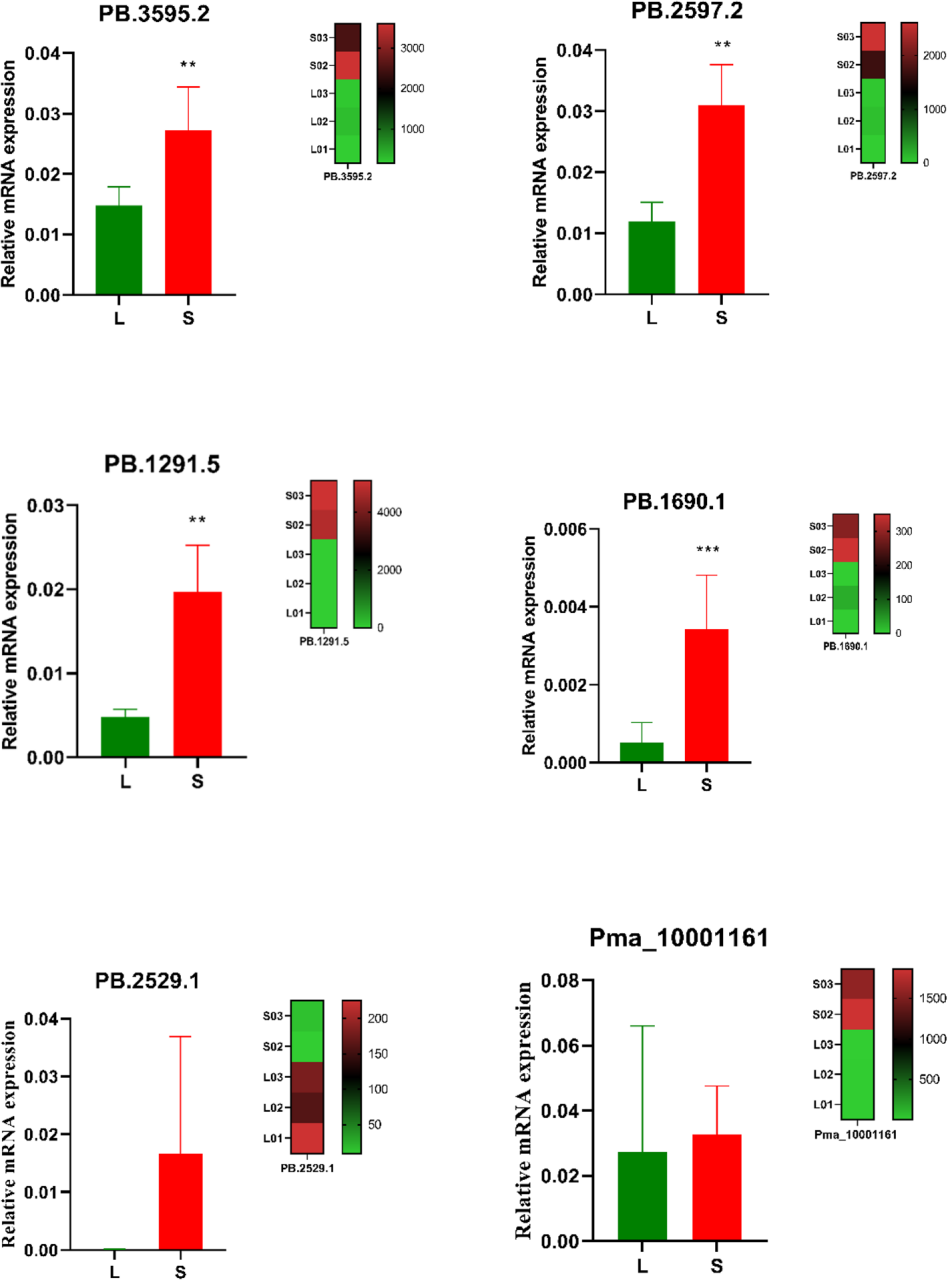
Relative mRNA expression profiles of six transcripts in *Pincata fucata martensii* selected for verification by quantitative real-time RT-PCR analysis. The number of transcripts in the transcriptome is shown in the heat map. Statistical changes were determined by Student’s t tests (two-tailed) and are denoted as ** *p* < 0.002 and ***p < 0.0002

Six transcripts were selected for validation by RT-qPCR. These transcripts were PB.2597.2 (proliferation-associated protein 2G4), PB.3595.2 (neural cell adhesion molecule 1), PB.1291.5 (monocarboxylate transporter 9), PB.1690.1 (cell division cycle 16-like protein), PB.2529.1 (fatty acid-binding protein) and Pma_10001161 (mineralisation-related protein 1). The RT-qPCR results showed that four transcripts (PB.2597.2, PB.3595.2, PB.1291.5 and PB.1690.1) were significantly up-regulated in S groups. However, the RT-qPCR and RNA-seq results for PB.2529.1 and Pma_10001161 were inconsistent. They did not show a significant difference by RT-qPCR (Fig. 8).

## Discussion

### PacBio sequencing can optimize genome structure

Due to the limitations of short read sequencing, annotation of the reference genome is often not sufficiently accurate. In our present work, a hybrid sequencing approach was used to optimise the genetic structure of the original annotation. The original boundary of 11,235 genes on the chromosomes was corrected. Additionally, 1607 gene loci were newly discovered in the *P. f. martensii* genome, and 14,946 transcripts were newly identified that were absent from the known transcriptome annotation. Thus, PacBio sequencing can be an effective strategy for improving the accuracy and quality *P. f. martensii* genome annotation information.

### PacBio sequencing reveals complexity and diversity in the *P. f. martensii* transcriptome

In eukaryotes, transcripts are highly complex and diverse since precursor mRNAs are subjected to multiple post-transcriptional modification processes, such as AS and APA [30, 31]. RNA-seq has showed that eukaryotic transcriptomes are highly complex due to post-transcriptional processing of precursor mRNAs by AS and APA [15, 32]. Herein, we identified AS and APA events from *P. f. martensii.* Differential polyadenylation of mRNAs generates transcript isoforms, and APA is necessary for RNA transport, localisation, stability and translation [30, 33]. Illumina sequencing has been widely used for gene expression analysis. However, it is unable to accurately detect APA, AS, fusion genes and gene families [34, 35]. The unigenes obtained in this study displayed high functional annotation rates of up to 82.26%. This may be attributed to the long-read sequences obtained using the PacBio single-molecule approach, which offers a considerable advantage for transcriptome-wide identification of full-length splice isoforms and other forms of post-transcriptional regulatory events such as APA [15]. PacBio sequencing technology can capture FL transcripts without the need for further assembly, making it more popular for transcriptome analysis [36, 37]. FL transcripts can also provide deep insight into transcriptome characterisation, and can greatly improve the accuracy of genome annotation [29]. However, PacBio sequencing has a higher error rate and must be corrected using Illumina sequencing reads [38] and/or CCS reads [39, 40]. By combining PacBio sequencing and Illumina sequencing, FL transcriptome data has great potential for the discovery of novel or previously unrecognised protein-coding genes and isoforms [41]. In the present work, 12,025 complete ORF sequences were obtained.

### Hybrid sequencing analysis reveals insight into the growth and development of *P. f. martensii*

In the present work, 228 DETs were identified between L and S groups, of which 99 DETs were up-regulated and 129 down-regulated in L groups. We analysed the mRNA expression profiles of six growth and development-related genes, and four of the six genes were up-regulated in S groups. Monocarboxylate transporter (MCT) is an important transmembrane transporter involved in many physiological functions, such as the regulation of nutrient absorption, intracellular pH, drug delivery and metabolism homeostasis [42–44]. Neuronal cell adhesion molecule 1 (NCAM 1) is a member of the immunoglobulin (Ig) superfamily of adhesion molecules, which are involved in binding between cells, helping cells stick to each other and their surroundings. NCAMs regulate bodyweight and energy homeostasis [45, 46]. Proliferation-associated 2G4 proteins are a conserved family in eukaryotes that are involved in cell proliferation and differentiation [47, 48]. Cell division cycle 16-like protein is one of the components of the anaphase-promoting complex involved in cell division [49]. Fatty acid-binding protein is an intracellular fatty acid carrier protein that plays an important role in the utilisation of fatty acids in cells [50]. Mineralization-related protein 1 is related to biomineralisation. Two of these transcripts were inconsistent between RT-qPCR and RNA-seq data. RNA-seq is a method of large-scale screening that reflects trends in gene expression in whole samples, but it does not guarantee that trends for each gene are consistent between RNA-seq and qPCR methods. Previous research reported that the majority of differentially expressed metabolites are up-regulated in small individuals [24, 51]. Additionally, some genes related to growth and development, shell formation, and reproduction are expressed more highly in S groups compared with L groups in pearl oyster [26]. This could be explained by the ‘catch-up growth’ phenomenon. Furthermore, immunisation may hinder growth [52]. Nutrients and energy ordinarily used for growth may be diverted to the generation of immune responses. Analysis of size in *Pinctada maxima* through transcriptomics indicated that low growth in small individuals could be partially explained by energy allocated for immune responses caused by stressors under certain environmental conditions [24]. In the present work, in addition to growth and development-related genes, some genes related to immune processes (PB.1364.11, PB.4993.4 and PB.3677.1) were differentially expressed between L and S groups. These results are in accordance with previous research [24]. Additionally, in our transcriptome databases, we identified numerous hypothetical proteins and uncharacterised protein displaying extremely significant differences. These proteins will be investigated in future research.

## Conclusion

We present the full-length transcriptome of *P. f. martensii* using PacBio single-molecule long-read sequencing. The results improve existing genome annotations, optimise the genome structure, and in-depth revealing the complexity and diversity of the differential growth patterns of *P. f. martensii* individuals. The findings may prove valuable for dissection mechanisms of growth traits and guiding molecular breeding in *P. f. martensii*.

## Methods

### Sample collection and RNA preparation

*Adult P. f. martensii* were obtained from the Daya Bay Marine Biology Research Stations of the Chinese Academy of Sciences (Shenzhen, Guangdong, P.R. China). Seven tissues including mantle, digestive gland, gonad, adductor muscle, foot, heart and gill were harvested and stored in liquid nitrogen until RNA extraction. Total RNA from each tissue was extracted and purified using TRIzol reagent following the manufacturer’s instructions (Magen, Guangzhou, China). RNA purity, concentration and integrity were assessed using an Agilent RNA 6000 nano kit and chips on a Bioanalyzer 2100 (Agilent Technologies, Santa Clara, CA, USA) and processed further for cDNA preparation. For PacBio sequencing, RNA samples from each tissue were mixed in an equal molarity for cDNA library construction. For Illumina sequencing, 100 one-year-old individuals were randomly selected from a Shenzhen population and shell length (SL) was measured, pearl oysters the top 9 and the bottom 9 in SL value were selected as largest individual (L group) and smallest individual (S group), respectively. The mantle tissue of each individual was collected and extracted for total RNA. RNAs from L group or S group were randomly divided into three subgroups, namely L01, L02, L03 or S01, S02, S03. Specifically, equal quantities of RNAs from three largest individual (L group) or smallest (S group) individual were pooled into one mixed sample for cDNA library construction.

### Illumina sequencing library construction and sequencing

Six double-stranded cDNA libraries were prepared using NEBNext® Ultra™ RNA Library Prep Kit according to the manufacturer’s methods. Briefly, mRNA was enriched from total RNA using Oligo (dT)-attached magnetic beads and randomly interrupted by fragmentation and reverse transcribed into double-stranded cDNA. The purified double-stranded cDNA was subjected to terminal repair and then ligated to sequencing adapters. Fragments of 380 ~ 530 bp were then selected, purified and subsequently PCR amplified to create the final cDNA library template for sequencing. The final library was quantitated using an Agilent 2100 Bioanalyzer instrument (Agilent). Six qualified libraries (L01, L02, L03; S01, S02, S03) were sequenced using an Illumina HiSeq 2000 System with pair-end 150 bp read length.

### PacBio sequencing library construction

mRNA was isolated from 5 μg of mixed total RNA using poly (T) oligo-attached magnetic beads. Fragmentation was treated with divalent cations under elevated temperatures in the NEBNext First Strand Synthesis Reaction Buffer (5×). Full-length cDNA was synthesized by SMARTer™ PCR cDNA Synthesis Kit (Clontech, CA, USA). Remaining overhangs were converted into blunt ends by exonuclease/polymerase activities. After adenylation of the 3′ ends of the DNA fragments, hybridization was performed by ligating NEBNext adaptor with a hairpin loop structure. The size selection of the full-length cDNA and for building libraries of differently sized cDNA were performed by BluePippin® (Sage Science, Beverly, MA, USA). The generated cDNA was then re-amplified using PCR, and the fragment size distribution was quantified using the Qubit fluorometer (Life Technologies, Carlsbad, CA, USA). The quality of the libraries was assessed using the Agilent Bioanalyzer 2100 system.

### PacBio sequencing processing

PacBio sequencing was performed using a PacBio RS II instrument. Raw reads were processed into error-corrected reads of inserts (ROIs) using the Iso-seq pipeline with full passes ≥0 and predicted accuracy > 0.80. Full-length transcripts were identified by searching for both 5′ and 3′ primer sequences and poly (A) tails in ROIs. Consensus isoforms and FL consensus sequences were obtained from Iterative Clustering for Error Correction using Quiver. High-quality FL transcripts were classified with post-correction accuracy > 9%. FL consensus sequences were mapped to the reference genome using Genomic Mapping and Alignment Program (GMAP) [53], and mapped reads were further collapsed by the pbtranscript-ToFU package with min-coverage = 85% and min-identity = 90%. Differences in 5′ sequences were not considered when collapsing redundant transcripts and Illumina data were used for PacBio sequencing error correction. The integrity of de-duplicated transcriptomes was assessed using BUSCO v3.0 [54].

### Illumina sequencing data processing

Raw data (raw reads) were firstly processed to remove adapters, reads containing poly-N, and low-quality reads, generating clean data (clean reads). At the same time, Q20, Q30, GC-content and sequence duplication values were calculated for clean data. All downstream analysis was based on clean data of high quality. Clean reads were then mapped to the reference genome sequence. Only reads with a perfect match or one mismatch were further analysed and annotated based on the reference genome. Tophat 2.1.1 (http://ccb.jhu.edu/software/tophat/index.shtml) was used to map to the reference genome.

### Prediction of fusion transcripts

The criteria for fusion candidates were (1) each transcript must be mapped to two or more distinct protein-encoding loci in the genome; (2) each mapped locus must align at least 5% of the transcript; (3) the combined alignment total coverage across all matched loci must be ≥99%; (4) each mapped locus is at least 10 kb apart.

### Structural analysis of transcripts

Transcripts were validated against known reference transcript annotations with the python library MatchAnnot (https://github.com/TomSkelly/MatchAnnot). Coding region sequences and their corresponding amino acid sequences were analysed using TransDecoder v3.0.0 (https://transdecoder.github.io/) software based on new transcripts obtained from alternative splicing. Transcription factors were identified using the animalTFDB 2.0 database [55]. Alternative polyadenylation (APA) analysis was conducted with TAPIS 1.2.1 (https://bitbucket.org/comp_bio/tapis/overview), and AS events including IR, ES, AD, AA and MEE were identified by the AStalavista tool 3.0 [56].

### Functional annotation and enrichment analysis

The functions of non-redundant transcripts were annotated using the following databases: NR, Pfam, KOG/COG/eggNOG, Swiss-Prot, KEGG and GO. Transcripts were subjected to enrichment analysis using GO implemented by the GOseq R packages based on the Wallenius non-central hyper-geometric distribution, which can adjust for gene length bias. KEGG is a database resource for understanding biological systems and high-level functions. KOBAS 2.0 software [57] was used to identify KEGG pathways enriched with differentially expressed transcripts (DETs). GO and KEGG pathway enrichment analyses were assessed by Fisher’s exact tests corrected with a false discovery rate (FDR) of 1%.

### Molecular marker identification

Transcripts that were > 500 bp in length were selected for SSR analysis with MIcroSAtellite identification tool (MISA v1.0) (http://pgrc.ipk-gatersleben.de/misa/misa.html). MISA can identify seven SSR types, namely mono-nucleotide, di-nucleotide, tri-nucleotide, tetra-nucleotide, penta-nucleotide, hexa-nucleotide, and compound SSR.

### Long-coding RNAs (lncRNAs) analysis

Putative protein-coding RNAs were filtered using minimum length and exon number thresholds. Non-protein-coding RNA transcripts with more than two exons and longer than 200 bp were sorted as lncRNA candidates using four computational approaches, namely Coding Potential Assessment Tool (CPAT), Coding-Non-Coding Index (CNCI), Coding Potential Calculator (CPC), and Pfam protein structure domain analysis (Pfam), all of which can distinguish protein-coding and non-coding transcripts. The identification of long-coding lncRNAs were chosen for predicting target genes based on location and co-expression relationship.

### Quantification of transcript expression levels and differential expression analysis

Unigene expression levels were analysed based on short read datasets generated by the Illumina sequencing platform. Transcript levels were estimated using the fragments per kilobase of transcript per million (FPKM) method mapped using HISAT 2.0 (https://ccb.jhu.edu/software/hisat2/index.shtml). Mantle tissue was used as the experimental tissue. Analysis of differential transcripts between two groups was performed using the DESeq R package (1.10.1) method [58]. DESeq provides statistical routines for determining differential expression in digital transcript expression data using a model based on the negative binomial distribution. The resulting *p*-values were adjusted using Benjamini and Hochberg’s approach for controlling the FDR. Only genes with an absolute value of |log2FC| ≥ 2 and FDR significance score < 0.01 were used for subsequent differentially expressed analysis.

### Quantitative real-time PCR analysis of DETs

DETs were selected to validate novel transcripts identified via RNA-seq. Five random individuals were sampled from largest or smallest groups, respectively. Total RNA was extracted from mantle tissue using TRIzol reagent and subsequently reverse-transcribed using a First-strand cDNA Synthesis Kit (Thermo Scientific, USA) following the manufacturer’s instructions. Primer sequences are listed in Additional file 11: Table S11. Real-time PCR was performed using a Roche LightCycler480 instrument (Roche, Basel, Switzerland) according to the manufacturer’s protocol. The reaction system consisted of 5 μl of SYBR Green (Toyobo), 0.3 μl of each primer (10 μM), 1 μl of diluted cDNA and 3.4 μl of ultrapure water to a total volume of 10 μl. PCR included an initial denaturation step at 95 °C for 30 s, followed by 40 cycles at 95 °C for 5 s, 57 °C for 10 s and 72 °C for 15 s. Each sample was tested in triplicate. The 18S ribosomal RNA gene was used as an internal control, and the comparative 2^-ΔΔCT^ method was used to analyze expression levels of candidate genes.

## Supplementary information

**Additional file 1: Table S1a, b.** Gene structure optimize.

**Additional file 2: Table S2a, b.** Transcripts and its corresponding genomic mapping data.

**Additional file 3: Table S3.** SSR analysis result statistics.

**Additional file 4: Table S4.** Alternative polyadenylation analysis result.

**Additional file 5: Table S5a, b.** Five kinds of alternative splicing events were identified.

**Additional file 6: Table S6.** The annotation of novel genes in database.

**Additional file 7: Table S7a, b.** LncRNAs were identified.

**Additional file 8: Table S8.** The predicting target genes of lncRNAs based on location and co-expression relationship.

**Additional file 9: Table S9.** Differential alternative splicing analysis between smallest (S) and largest (L) subgroup.

**Additional file 10: Table S10.** The differentially expression transcript analysis data.

**Additional file 11: Table S11.** The nucleotide sequences of the primers for PCR.

## Data Availability

The raw sequence data reported in this paper have been deposited in the Genome Sequence Archive in National Genomics Data Center, Beijing Institute of Genomics, Chinese Academy of Sciences, under accession number CRA002619 that are publicly accessible at http://bigd.big.ac.cn/gsa. Data supporting the manuscript are also available in the supplementary information. The reference genome are used via the GigaDB database [59].

## Abbreviations

RNA: Ribonucleic acid
PCR: Polymerase chain reaction
SE: Skipped exon
MXE: Mutually exclusive exon
A5SS: Alternative 5′ splice site
A3SS: Alternative 3′ splice site
RI: Retained intron
