# Supplementary material for: PacBio single molecule long-read sequencing provides insight into the complexity and diversity of the Pinctada fucata martensii transcriptome
Source: BMC Genomics. 2020 Jul 13;21:481. doi: 10.1186/s12864-020-06894-3 (PMC7359550; doi:10.1186/s12864-020-06894-3)
Supplement: Supplementary file 9 — Additional file 9: Table S9. Differential alternative splicing analysis between smallest (S) and largest (L) subgroup. [file 12864_2020_6894_MOESM9_ESM.docx]

Additional file 9: Table S9 Differential alternative splicing analysis between smallest (S) and largest (L) subgroup

| EventType | NumEvents.JC.only | SigEvents.JC.only | NumEvents.JC+readsOnTarget | SigEvents.JC+readsOnTarget |
| --- | --- | --- | --- | --- |
| SE | 5095 | 83(33:50) | 5146 | 87(33:54) |
| MXE | 924 | 44(15:29) | 931 | 42(15:27) |
| A5SS | 251 | 4(1:3) | 252 | 4(1:3) |
| A3SS | 246 | 3(2:1) | 246 | 5(2:3) |
| RI | 171 | 10(1:9) | 175 | 9(1:8) |

NumEvents.JC. only: total number of events detected using Junction Counts only.

SigEvents.JC. only: number of significant events detected using Junction Counts only.

NumEvents.JC+readsOnTarget: total number of events detected using both Junction Counts and reads on target.

SigEvents.JC+readsOnTarget: number of significant events detected using both Junction Counts and reads on target.

The numbers in the parentheses (n1:n2) indicate the number of significant events that have higher inclusion level for subgroup S (n1) or for subgroup L (n2).

SE: Skipped exon

MXE: Mutually exclusive exon; A5SS: Alternative 5' splice site; A3SS: Alternative 3' splice site; RI: Retained intron.
