# Supplementary material for: PacBio single molecule long-read sequencing provides insight into the complexity and diversity of the Pinctada fucata martensii transcriptome
Source: BMC Genomics. 2020 Jul 13;21:481. doi: 10.1186/s12864-020-06894-3 (PMC7359550; doi:10.1186/s12864-020-06894-3)
Supplement: Supplementary file 11 — Additional file 11: Table S11. The nucleotide sequences of the primers for PCR. [file 12864_2020_6894_MOESM11_ESM.docx]

Additional file 11: Table S11

Nucleotide sequences of the primers for PCR

| Target genes | Primers sequences(5’-3’) |
| --- | --- |
| PB.1690.1-F | TCATGGTTTGCCGTAGGGTG |
| PB.1690.1-R | CGAGTGTCCAAATGCCAACC |
| PB.3595.2-F | GAGCCGTCCACAGCCTATAC |
| PB.3595.2-R | TCACGCTGACCTCATATGGC |
| PB.2597.2-F | AGCACGACTTGATGTCACCC |
| PB.2597.2-R | CGGTAAGCCGGTGATCTTGA |
| PB.1291.5-F | GGAGGCCGCTTTCTTAGTGT |
| PB.1291.5-R | GTCAGCCATCCATCCGAACA |
| PB.2529.1-F | TAACGAGAGAGGTGACGGG |
| PB.2529.1-R | ATACTGTGTGACAGGCTCGG |
| Pma_10001161-F | CCGCCTCCTACTGGACAAAG |
| Pma_10001161-R | ATCTGTTTGGCTCTCGCACA |
| 18sF | CGTTTCAACAAGACGCCAGTAG |
| 18sR | ACGAAAAAAAGGTTTGAGAGACG |
